# Identification and characterization of RNA guanine-quadruplex binding proteins

**DOI:** 10.1093/nar/gku290

**Published:** 2014-04-25

**Authors:** Annekathrin von Hacht, Oliver Seifert, Marcus Menger, Tatjana Schütze, Amit Arora, Zoltán Konthur, Peter Neubauer, Anke Wagner, Christoph Weise, Jens Kurreck

**Affiliations:** 1 Institute of Biotechnology, Department of Applied Biochemistry, TUB 4/3–2, Technische Universität Berlin, Gustav-Meyer-Allee 25, 13355 Berlin, Germany; 2 Institute for Cell Biology and Immunology, University of Stuttgart, Allmandring 31, 70569 Stuttgart, Germany; 3 RiNA GmbH, Volmerstraße 9, 12489 Berlin, Germany; 4 Institute for Molecular Biosciences, University of Frankfurt, Max-von-Laue-Str. 9, 60438 Frankfurt/Main, Germany; 5 Max Planck Institute for Molecular Genetics, Ihnestraße 63–73, 14195 Berlin, Germany; 6 Max Planck Institute of Colloids and Interfaces, Am Mühlenberg 1, 14476 Potsdam, Germany; 7 Institute of Biotechnology, Department of Bioprocess Engineering, ACK-24, Technische Universität Berlin, Ackerstraße 76, 13355 Berlin, Germany; 8 Institute of Chemistry and Biochemistry, Freie Universität Berlin, Thielallee 63, 14195 Berlin, Germany

## Abstract

Guanine quadruplex (G-quadruplex) motifs in the 5′ untranslated region (5′-UTR) of mRNAs were recently shown to influence the efficiency of translation. In the present study, we investigate the interaction between cellular proteins and the G-quadruplexes located in two mRNAs (MMP16 and ARPC2). Formation of the G-quadruplexes was confirmed by biophysical characterization and the inhibitory activity on translation was shown by luciferase reporter assays. In experiments with whole cell extracts from different eukaryotic cell lines, G-quadruplex-binding proteins were isolated by pull-down assays and subsequently identified by matrix-assisted laser desorption ionization-time of flight mass spectrometry. The binding partners of the RNA G-quadruplexes we discovered included several heterogenous nuclear ribonucleoproteins, ribosomal proteins, and splicing factors, as well as other proteins that have previously not been described to interact with nucleic acids. While most of the proteins were specific for either of the investigated G-quadruplexes, some of them bound to both motifs. Selected candidate proteins were subsequently produced by recombinant expression and dissociation constants for the interaction between the proteins and RNA G-quadruplexes in the low nanomolar range were determined by surface plasmon resonance spectroscopy. The present study may thus help to increase our understanding of the mechanisms by which G-quadruplexes regulate translation.

## INTRODUCTION

DNA and RNA molecules rich in guanine have the potential to form unusual secondary structures known as guanine quadruplexes (G-quadruplexes). These motifs comprise a structure of π-stacked tetrads formed by the coplanar arrangement of four Hoogsteen-paired guanines (1). The stability of G-quadruplex structures is significantly affected by the presence of a central cation, with potassium being the most suitable ion to stabilize G-quadruplexes (2). G-quadruplex motifs in DNA have been studied extensively for many years. For example, cellular G-quadruplexes in the telomeres have been shown to be important for the maintenance of eukaryotic chromosomes (3). Furthermore, G-quadruplex motifs in the promoter region of various genes involved in carcinogenesis are considered to control gene expression on the transcriptional level (for a review, see (4)). Recently, the existence of G-quadruplexes in human cells was visualized for the first time with a structure-specific antibody (5). Since RNA is usually single-stranded, the formation of G-quadruplex structures can be expected to occur even more easily than in double-stranded DNA, as base-pairing with complementary sequences will not compete with the folding process. Research in the last few years revealed that G-quadruplexes in RNA molecules fulfill a number of important cellular functions: telomeric repeat-containing RNA (TERRA) was described to regulate telomerase activity and further RNA G-quadruplexes are involved in termination of transcription, polyadenylation, regulation of alternative splicing, subcellular sorting of mRNAs and modulation of translation (2,6–9).

Of particular interest for the present study is the finding that G-quadruplexes in the 5′-UTRs of mRNAs interfere with translation. In 2007, Balasubramanian and coworkers (10) first reported that the presence of an RNA G-quadruplex in the 5′-UTR of an mRNA exhibits an inhibitory effect on translation. While these experiments were carried out in an *in vitro* translation assay, we were able to demonstrate that the evolutionarily conserved G-quadruplex in the mRNA of the zinc-finger protein of the cerebellum 1 (Zic-1) drastically inhibits translation in human cells without altering the mRNA level (11). Thus, the G-quadruplex only regulates the translation process and does not significantly influence transcription. Further experiments confirmed that the Zic-1 protein was only synthesized in the absence of a 5′-UTR, whereas a 73 nucleotide-long fragment of the 5′-UTR containing the G-quadruplex motif inhibited expression of *Zic-1*.

A bioinformatic analysis carried out by Huppert et al. (12) revealed that around 2000 genes of the human genome contain potential G-quadruplexes in the 5′-UTR and that the G-quadruplex motifs are overrepresented at the 5′-end of the 5′-UTRs. In a systematic approach, Halder et al. analyzed the influence of loop size and number of GGG-repeats and observed a correlation between the inhibitory activity of G-quadruplexes and their stability, which depends on the number of nucleotides in the loop and repeat numbers (13). While the initial studies used isolated G-quadruplex motifs as a 5′-UTR, subsequent experiments confirmed the inhibitory effect of a G-quadruplex in the natural, full-length 5′-UTR of the matrix metalloproteinase *MT3-MMP*, also known as *MMP16* (14). Partial repression of translation by G-quadruplexes in the 5′-UTR of mRNAs has, in the meantime, been reported for various other genes such as the *fragile X mental retardation protein* (*FMRP*), *Telomeric repeat-binding factor 2* (*TRF2)*, the *human estrogen receptor α* and *B-cell lymphoma 2 (Bcl-2)* (15–18). In an attempt to investigate the biological relevance of G-quadruplexes, such an element in the 5′-UTR of the cyclin D3 mRNA was shown to affect cell cycle progression (19). Surprisingly, a G-quadruplex in the 5′-UTR of the *Transforming Growth Factor-beta (TGF-β2)* was recently reported to augment translation (20). Furthermore, a G-quadruplex motif in the 3′-UTR of the proto-oncogene *Pim1* was reported to repress translation in a similar manner as the majority of the G-quadruplexes in the 5′-UTR (21). For a complete survey of RNA G-quadruplexes in mRNAs, see (5).

The exact mechanism of the translational inhibition by G-quadruplex motifs in the 5′-UTR has not yet been elucidated. In the absence of evidence for a more complex mechanism, it is reasonable to hypothesize that the stable structure sterically blocks the ribosome. Most of the G-quadruplex motifs in the 5′-UTRs of mRNAs were found to repress translation by approximately 50% and only a few cases have been reported to date, in which a stronger inhibitory effect of up to 80% has been observed (2,22). It is therefore likely that an equilibrium exists between the folded state, in which the G-quadruplex is formed and translation is prevented, and an open state, which allows the ribosome to perform protein synthesis.

A number of small molecular compounds have been characterized that modulate the stability of G-quadruplexes. The porphyrin TmPyP4 (5,10,15,20-tetra(*N-*methyl-4-pyridyl)porphyrin) was found to stabilize the DNA G-quadruplex of the c-myc promoter, thereby repressing transcription (23). Interestingly, an opposite effect was observed for G-quadruplexes composed of RNA: TmPyP4 unfolds the extremely stable G-quadruplex of *MMP16* and alleviates its repressive effect (24). As a consequence, translation of the coding sequence downstream of the G-quadruplex was enhanced. In a similar manner, the destabilizing effect of TmPyP4 on the G-quadruplex of the *fragile X premutation* mRNA resulted in an increase of translation (25). In contrast, small molecules such as bisquinolinium compounds and a pyridine-2,6-bis-quinolino-dicarboxamide derivative named RR110 were found to stabilize G-quadruplexes in the mRNAs, thereby repressing translation (26,27).

Several studies have addressed interactions between proteins and G-quadruplexes in telomeres and promoters. For example, affinity purification assays revealed the multifunctional phosphoprotein nucleolin to interact with the DNA G-quadruplex in the c-myc promoter (28). Binding of nucleolin was found to stabilize the G-quadruplex structure and to reduce the promoter activity. While considerable knowledge has been accumulated about the interaction between DNA G-quadruplex motifs and proteins, only very little is known about protein binding to G-quadruplex-forming RNA sequences (29). On the RNA level, FMRP was described to bind to a G-quadruplex motif in the coding region of its own mRNA, thereby inhibiting translation by a negative feedback loop (30). Furthermore, pull-down assays, followed by matrix-assisted laser desorption ionization-time of flight (MALDI-TOF) mass spectrometry (MS), were used to identify proteins that interact with the G-rich TERRA (31). Among the proteins that bind to the TERRA transcript were members of the heterogenous nuclear ribonucleoprotein (hnRNP) family. To the best of our knowledge, no studies have been carried out to date to identify proteins that bind to the G-quadruplex motifs in 5′-UTRs.

The aim of the present study was therefore to characterize interactions between cellular proteins and G-quadruplexes located in the 5′-UTRs of mRNAs. We chose two factors whose protein levels were shown to play a role in tumor pathology. MMP16 is a member of the family of matrix metalloproteinases that are involved in the degradation of extracellular matrix proteins. Upregulation of MMP16 has been described to be associated with the invasiveness of cancer cell (32) and angiogenesis in tumors (33). The second factor under investigation, the actin-related protein 2 (ARPC2), is one of the seven subunits of the ARP2/3 protein complex, which is involved in actin cytoskeleton branching and thereby contributes to cell migration. While cell migration is essential in several physiological biological processes, its aberrant activation may also lead to the formation of metastases in cancer. Silencing of the ARP2/3 complex was recently shown to disturb pancreatic cancer cell migration (34). To fully understand their relevance in tumor biology, it is thus important to elucidate regulation mechanisms that influence the protein levels of these two factors.

## MATERIALS AND METHODS

### Cell culture

HeLa and HEK293 cells were cultured in low glucose Dulbecco's modified Eagle's medium (PAA Laboratories GmbH, Cölbe, Germany) containing 10% fetal bovine serum, 2 mM glutamine, non-essential amino acids and the antibiotics penicillin and streptomycin. HEK293 medium was supplemented with 4.5 mg/ml glucose. Cells were grown at 37°C in a humidified atmosphere with 5% CO_2_.

### Synthetic RNA Oligonucleotides

The G-quadruplex-forming sequences of the MMP16 and ARPC2 RNAs and their mutated controls were purchased from Purimex GmbH, Grebenstein, Germany:

MMP16 GQ, AACGAGGGAGGGAGGGAGAGGGAGAGA-biotin
MMP16 mut, AACGAGAGAGAGAGAGAGAGAGAGAGA-biotin
ARPC2 GQ, AGCCGGGGGCUGGGCGGGGACCG GGCUUGU-biotin
ARPC2 mut, AGCCGUAGACUGAGCGAAGACCGAGCUUGU-biotin
ARPC2–2xG, AGCCGUAGACUGGGCGAAGACCGGGCUUGU-biotin.

All oligonucleotides were biotinylated at their 3′ ends and polyacrylamide gel electrophoresis (PAGE) purified. The underlined nucleotides represent the core quadruplex forming sequences. MMP16 mut and ARPC2 mut were used to exclude proteins with a general RNA-binding capacity, while ARPC2–2xG was included to identify proteins that preferentially bind to G-rich sequences rather than G-quadruplex structures.

### Quadruplex formation

Quadruplex formation was carried out by dissolving biotinylated RNA oligonucleotides in folding buffer containing 10 mM Tris-HCl, pH 7.5, 100 mM KCl and 0.1 mM EDTA. Formation of secondary structures was achieved by heating the RNA to 95°C for 5 min in a thermocycler and cooling it down to 4°C in 2°C per minute steps.

### CD and UV spectroscopy

Prior to CD experiments, quadruplex formation of 1 μM RNA was carried out. Circular Dichroism (CD) experiments were performed at 20°C using a JASCO J-810 spectropolarimeter (JASCO, Gross-Umstadt, Germany) equipped with a Peltier temperature controller. Spectra were recorded in 1 mm quartz cuvettes. CD scans were taken from 220 to 320 nm in duplicates each with four accumulations and their average was calculated. A CD spectrum of the buffer was recorded and subtracted from the spectrum obtained for the RNA-containing solution.

For UV melting and annealing studies, RNA samples were prepared at 1 μM concentration in 10 mM Tris-HCl, pH 7.5 with KCl (10 mM and 100 mM). UV annealing and melting studies were carried out on a JASCO V-650 UV–visible spectrophotometer equipped with a Peltier temperature controller. Samples were heated to 95°C and cooled down to 20°C at a 0.2°C min^−1^ temperature gradient, and absorption data recorded at 295 nm were collected every 0.5 min on both annealing and melting steps. The wavelength of 295 nm was previously shown to yield more precise results than 260 nm when studying G-quadruplexes (35,36). The annealing and melting cycles were performed in duplicate in three independent experiments.

### RT-PCR experiments

Total cellular RNA was extracted from HeLa and HEK293 cells using the RNeasy mini kit (QIAGEN, Hilden, Germany). To remove DNA contaminations, DNase digestion was performed with RQ1 RNase-Free DNase (Promega, Mannheim, Germany). cDNA was synthesized with Revert Aid M-MuLV Reverse Transcriptase (Fermentas, St. Leon-Rot, Germany) and random hexamer primers from 1 μg RNA template. Polymerase chain reaction (PCR) samples contained 1 Unit Taq Polymerase (Rapidozym, Berlin, Germany), 0.2 mM dNTPs, 1 μl cDNA template and 1 μM of each primer. The primer pairs MMP16 fw/rev or ARPC2 fw/rev were used:

MMP16 fw, AAAATGGCAAACGTGATGTGG
MMP16 rev, GGCTCATCTGAGTCAAAATGG
ARPC2 fw, AGAAGAGGGCAAGGAAGGAG
ARPC2 rev, TGGCTAAAGAGGACCTGTGG.

Amplification was performed for 30 cycles. PCR products were separated on 1.5% agarose gels and visualized by ethidium bromide staining.

### Dual luciferase assays

To construct the plasmids psiCHECK-2-MMP16-GQ, psiCHECK-2-MMP16-mut, psiCHECK-2-ARPC2-GQ and psiCHECK-2-ARPC2-mut, the following DNA oligonucleotides containing NheI restriction sites were used:

MMP16 GQ sense CTAGAACGAGGGAGGGAGGGAGAGGGAGAGA
MMP16 GQ antisense CTAGTCTCTCCCTCTCCCTCCCTCCCTCGTT
MMP16 mut sense CTAGAACGAGAGAGAGAGAGAGAGAGAGAGA
MMP16 mut antisense CTAGTCTCTCTCTCTCTCTCTCTCTCTCGTT
ARPC2 GQ sense CTAGAGCCGGGGGCTGGGCGGGGACCGGGCTTGT
ARPC2 GQ antisense CTAGACAAGCCCGGTCCCCGCCCAGCCCCCGGCT
ARPC2 mut sense CTAGAGCCGTAGACTGAGCGAAGACCGAGCTTGT
ARPC2 mut antisense CTAGACAAGCTCGGTCTTCGCTCAGTCTACGGCT.

Each pair of sense and antisense oligonucleotide was annealed and ligated into the unique NheI restriction site of the psiCHECK-2 plasmid (Promega) upstream of the *Renilla* luciferase start codon.

For the dual luciferase assay, subconfluent HEK293 cells in 24-well plates were transfected with 0.8 μg of psiCHECK-2 reporter plasmids described above with Lipofectamine 2000 (Life Technologies GmbH, Darmstadt, Germany) according to the manufacturer's instructions. Activities of firefly and *Renilla* luciferase were measured 24 h after transfection using the Dual-Luciferase Reporter Assay Kit (Promega) on a luminometer (Lumat LB, Berthold, Pforzheim, Germany).

### Pull-down assays and MALDI-TOF mass spectrometry

For the preparation of whole cell extracts, confluent HeLa or HEK293 cell cultures were harvested by trypsination, washed with phosphate buffered saline (PAA Laboratories GmbH) and resuspended in one volume of buffer A (10 mM Tris-HCl, pH 7.5, 10 mM KCl, 1.5 mM MgCl_2_, 0.5 mM dithiothreitol (DTT)). After 15 min incubation on ice, cells were pelleted and resuspended in two volumes buffer A and one volume buffer B (300 mM Tris-HCl, pH 7.5, 1.4 M KCl, 3 mM MgCl_2_) containing protease inhibitor (Complete, Roche, Mannheim, Germany). Cells were disrupted by sonification and insoluble cell matter was removed by centrifugation (10 000 x g, 20 min, 4°C). The resulting protein extract was dialyzed against buffer C (20 mM Tris-HCl, pH 7.5, 100 mM KCl, 20% (v/v) glycerol, 0.5 mM DTT, 0.2 mM Na-EDTA) overnight at 4°C.

The whole cell extracts were used for pull-down assays with the G-quadruplex-forming RNA oligonucleotides. To avoid unspecific binding, streptavidin agarose beads (Sigma-Aldrich Chemie GmbH, Taufkirchen, Germany) were initially incubated in blocking buffer containing 10 mM Tris-HCl, pH 7.5, 100 mM KCl, 0.1 mM Na-EDTA, 1 mM DTT, 0.01% (v/v) Triton X-100, 0.1% (w/v) bovine serum albumin and 0.02% (w/v) tRNA from *Saccharomyces cerevisiae* (Sigma-Aldrich) for 1 h at 4°C on a rotating wheel. Folded 3′-biotinylated RNA oligonucleotides were coupled to 40 μl of the solution containing the streptavidin agarose beads at 4°C overnight. 400 μl of the protein extract was then incubated with streptavidin agarose beads for 1 h at 30°C. Bound proteins were eluted from the RNA by washing the beads with increasing KCl concentrations (400–2600 mM). Each fraction was concentrated by trichloroacetic acid precipitation and analyzed on a 10% sodium dodecyl sulphate-polyacrylamide gel electrophoresis (SDS-PAGE), stained with colloidal Coomassie staining as described in (37).

Peptides were obtained by trypsin in-gel digestion as described previously (38) and peptide masses were analyzed by MALDI-TOF MS using an Ultraflex-II TOF/TOF instrument (Bruker Daltonics, Bremen, Germany) equipped with a 200 Hz solid-state Smart beam™ laser. The mass spectrometer was operated in the positive reflector mode. Mass spectra were acquired over an m/z range of 600–4000. α-cyano-4-hydroxycinnamic acid was used as the matrix and protein digest samples were spotted using the dried droplet technique. MS/MS spectra of selected peptides were acquired in the LIFT mode (39). Database searches were performed using Mascot (Matrix Science Ltd., http://www.matrixscience.com). Mass tolerance was set at ± 75 ppm unless otherwise indicated (Supplementary Table S1) and we allowed for one missed cleavage.

### Cloning, expression and purification of recombinant proteins

For expression of recombinant proteins, human cDNA sequences were cloned into the pET28a expression vector (Merck, Darmstadt, Germany) using the BamHI and NdeI restriction sites, so that the expressed proteins contained an N-terminal His-Tag. The following oligonucleotides were used to amplify the coding sequences of nucleolin, EFHD2, SRSF1 and U2AF65:

Nucleolin Δ1–283 fw, GGAATTCCATATGGCCAAACAGAAAGCAGC
Nucleolin rev, GCGGGATCCCTATTCAAACTTCGTCTTC
EFHD2 fw, GGAATTCCATATGGCCACGGACGAGC
EFHD2 rev, GTAGGATCCTCAGGCTCGGCCTCC
SRSF1 fw, GGAATTCCATATGTCGGGAGGTGGTG
SRSF1 rev, GCGGGATCCTTATGTACGAGAGCGA
U2AF65 fw, GGAATTCCATATGTCGGACTTCGACGAG
U2AF65 rev, GTAGGATCCCTACCAGAAGTCCCGG.

Nucleolin was expressed as a variant consisting of amino acid residues 284–709 (in the following denoted as Δnucleolin), including all four RNA binding domains (RBDs) and the C-terminal domain.

Proteins were expressed in *Escherichia coli* BL21 DE3 using EnPresso^®^ B tablet medium (BioSilta, Oulu, Finland) according to the manufacturer's instructions at 30°C. Expression was induced with 0.2 mM final concentration isopropyl β-D-1-thiogalactopyranoside and cells were harvested 24 h after induction. Cell pellets were harvested by centrifugation (20 min, 4000 x g), resuspended in lysis buffer (50 mM NaH_2_PO_4_, pH 8, 300 mM NaCl, 10 mM imidazole, 2 M NaCl, 2% Triton X-100, 5% glycerol) and disrupted by sonification. Crude extract was cleared by centrifugation for 30 min at 10 000 g. Proteins were purified with Protino® Ni-NTA Agarose (Macherey-Nagel, Düren, Germany) in gravity flow columns. Non-bound proteins were removed by washing with 50 mM NaH_2_PO_4_, pH 8, 300 mM NaCl, 20 mM imidazole, 2 M NaCl, 2% Triton X-100, 5% glycerol. Proteins were eluted in 50 mM NaH_2_PO_4_, pH 8.0, 300 mM NaCl, 250 mM imidazole, 2 M NaCl, 2% Triton X-100, 5% glycerol and dialyzed against 10 mM HEPES, pH 7.5, 150 mM NaCl, 10% glycerol, 1 mM DTT and 0.1% Triton X-100. Protein concentrations were determined using the Bicinchoninic Acid (BCA) Protein Assay Reagent (Thermo Fisher Scientific, Bonn, Germany).

### Surface plasmon resonance

Surface plasmon resonance (SPR) experiments were performed on the Biacore-X or Biacore 2000 platform (GE Healthcare Europe, Freiburg, Germany). All experiments were performed at 25 °C. Proteins were immobilized on carboxymethyldextran (CMD 500 L) sensor chips (Xantec, Duesseldorf, Germany) using amine coupling. Immobilization was performed in the SPR running buffer HBS-EP (0.01 M HEPES pH 7.4, 0.15 M NaCl, 3 mM EDTA, 0.005% v/v Surfactant P20; GE Healthcare Europe) using a flow rate of 5 μl/min. The surface of the CMD 500 chip was activated with 35 μl 0.02 M 1-ethyl-3-(3-dimethylaminopropyl)-carbodiimide and 0.05 M N-hydroxysuccinimide. Each Protein diluted in HBS-EP buffer was injected until around 1000 response units were immobilized, the surface was blocked with 35 μl 1 M ethanolamine (pH 8.5). An activated and blocked channel without protein served as reference. Binding analysis was conducted at a flow rate of 20 μl/min in a 1:10 mixture of binding buffer (10 mM Tris-HCl, pH 7.5, 150 mM KCl) and HBS-EP to match the buffer in which the oligonucleotides were diluted and to minimize bulk shift effects. Both channels of the flow cell were injected with 30 μl of the RNA solutions in a range of 6–1000 nM. After each RNA injection, the chip surface was regenerated by injection of 10 μl 1 M KCl. For background correction, each response signal was adjusted by subtraction of the reference cell signal as well as by subtraction of adjusted buffer injection signals. Association and dissociation rates and constants of the RNA-protein complexes were determined using BIAevaluation software (GE Healthcare Europe). The data were globally fitted to a 1:1 interaction model (Langmuir binding).

## RESULTS

### Biophysical characterization of the MMP16 and ARPC2 RNA G-quadruplexes

Two G-quadruplex motifs were chosen to study the interaction between RNA structures and cellular proteins. The G-quadruplex located in the 5′-UTR of MMP16, also known as MT3-MMP, was previously shown to form a very stable structure (14). In contrast, the G-quadruplex in the 5′-UTR of the mRNA of ARPC2 was only predicted by bioinformatics means (12) and has not yet been experimentally verified.

The two RNA G-quadruplex motifs were analyzed by the software quadruplex-forming G-Rich Sequences (QGRS) Mapper (http://bioinformatics.ramapo.edu/QGRS/index.php). QGRS Mapper is a software program that generates information about composition and distribution of putative QGRS and provides a G-Score as measurement of G-quadruplex probability (40). Both RNA sequences are able to form very stable G-quadruplex structures with three guanine tetrads. The ARPC2 G-quadruplex can form up to four variants of the stable structure with a G-Score of 39 and 41 and nucleotide length of 19 and 20 nt, while the MMP16 G-quadruplex can form only one variant with a G-Score of 40 and nucleotide length of 17 nt.

For pull-down assays, biotinylated oligonucleotides were used that consisted of the G-quadruplex-forming sequences and flanking nucleotides, in order to simulate the natural environment of the motif and to have a spacer between the biotin and the G-quadruplex motif. In the control oligonucleotides, several guanosines were substituted so that they cannot fold into a G-quadruplex conformation. These controls were used to exclude proteins with a general RNA-binding capacity. An additional control with a G-rich sequence (ARPC2–2xG) was used to identify proteins that bind to G-stretches but are not specific for G-quadruplex motifs.

**Figure 1.**
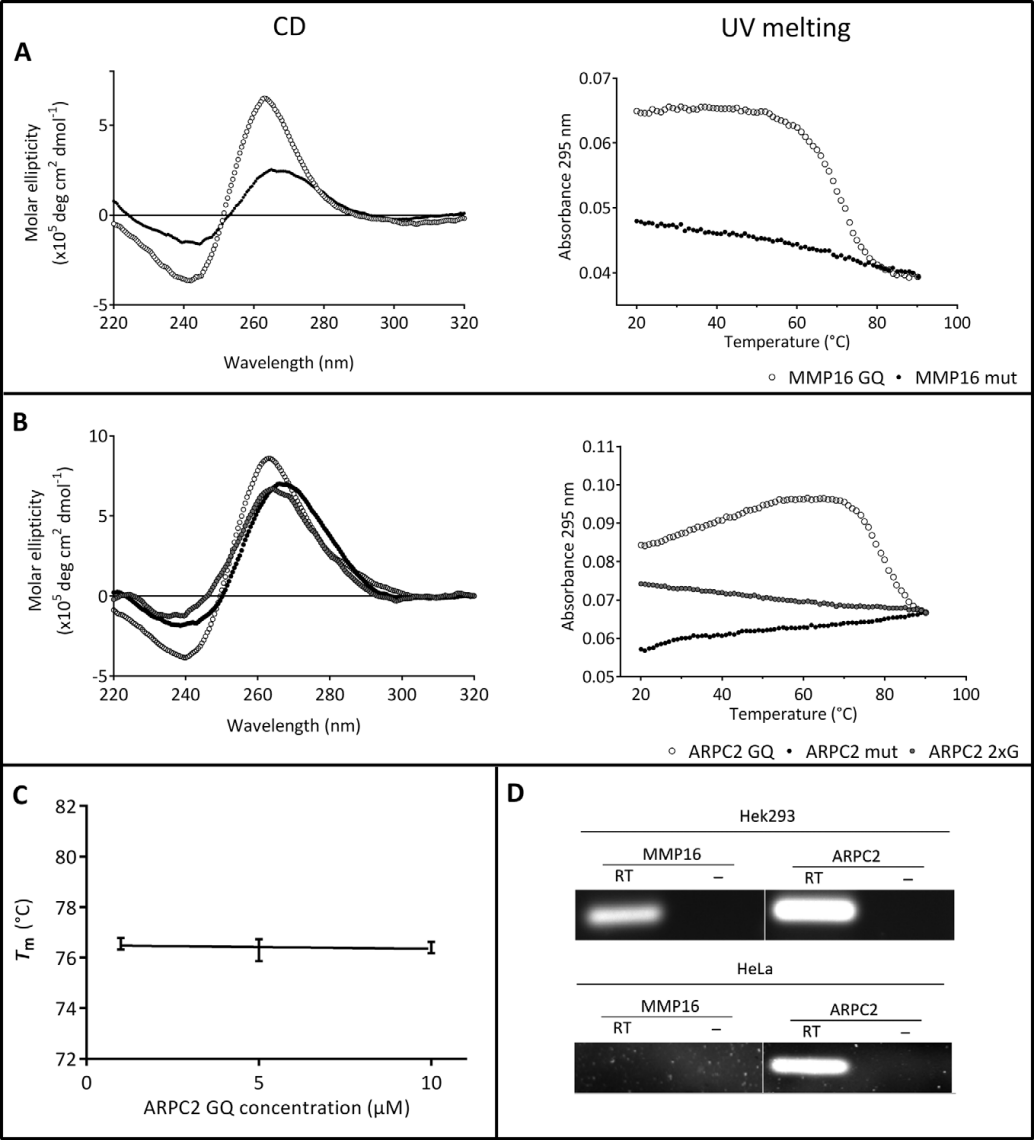
Basic characterization of the G-quadruplex motifs under investigation. (**A**) and (**B**) CD spectra and melting curves of the G-quadruplex motifs and their mutated control oligonucleotides (A: MMP16; B: ARPC2). (**C**) Melting temperature at different concentrations of the ARPC2 G-quadruplex. All melting experiments were carried out at 100 mM KCl, except for the MMP16 G-quadruplex-forming sequence, since it did not unfold in the measured temperature range. D) RT-PCR experiments to confirm expression of MMP16 and ARPC2 in HEK293 cells. Additional experiments were carried out with HeLa cells. Control experiments without reverse transcription are shown (–).

The first step was to analyze the formation of the G-quadruplex structure of the respective oligonucleotides by CD and UV spectroscopy. CD spectra of the G-rich MMP16 RNA sequence in 10 mM KCl showed a positive peak at 267 nm and a negative peak at 245 nm (Figure 1A) indicating the presence of a parallel G-quadruplex structure (10,41). The mutated variant lacks these characteristics since it cannot form a G-quadruplex structure. The CD data are in line with a previous report on this G-quadruplex (14). UV melting experiments carried out at 10 mM KCl showed a reversible sigmoidal transition at 295 nm (Figure 1A) indicative of the formation of a G-quadruplex structure (35). The *T*_m_ was found to be 69.2 ± 0.2°C at a KCl concentration of 10 mM. At higher salt concentrations (100 mM KCl) the structure could not be unfolded, demonstrating the high stability of the MMP16 RNA G-quadruplex (data not shown). No distinct transition was observed for the control oligonucleotide, as would be expected if it does not form a G-quadruplex.

For the G-rich oligonucleotide of the ARPC2 mRNA, the CD spectra showed a positive peak at 267 nm and a negative peak at 238 nm (Figure 1B), which again is indicative of the presence of a parallel G-quadruplex structure. A slight deviation from other reported values (10,11,41) can be due to the presence of flanking bases on either side of the core G-quadruplex-forming sequence that may form alternative structures. The CD spectra of the mutated controls (ARPC2 mut and ARPC2–2xG) showed positive peaks around 270 nm and negative peaks around 230–240 nm. As the spectra of the mutated sequences do not differ significantly from the CD spectrum of the putative G-quadruplex-forming sequence, UV melting analyses at 295 nm in 100 mM KCl were carried out to gain further information (Figure 1D). These experiments showed a sigmoidal transition with a *T*_m_ of 76.5 ± 0.2°C for the G-rich sequence of ARPC2, as expected for a G-quadruplex motif. Neither of the mutated controls showed a sigmoidal transition, indicating so that they do not to fold into a G-quadruplex structure.

Since the ARPC2 G-quadruplex has not been previously described, we characterized it further by measuring the concentration dependency of *T*_m_. As can be seen in Figure 1C, *T*_m_ does not vary in the concentration range under investigation. This finding further supports the hypothesis that the sequence forms an intramolecular G-quadruplex.

Taken together, all data obtained from UV melting and CD experiments are in good agreement with well-known characteristics for parallel G-quadruplex motifs. It is therefore reasonable to assume that both RNA oligonucleotides form G-quadruplex structures. The G-quadruplex of the ARPC2 G-rich sequence, however, seems to be less stable than that of the MMP16 G-quadruplex since it has to compete with alternative conformations.

### Expression of MMP16 and ARPC2 in HEK293 cells

In order to obtain biologically relevant data, pull-down assays were carried out with a cell line that endogenously expresses the two genes under investigation. Reverse transcriptase-PCR (RT-PCR) experiments confirmed that HEK293 cells express MMP16 as well as ARPC2. This cell line thus represents an orthotopic model to study the interaction between the mRNAs of MMP16 and ARPC2, respectively, and cellular proteins. Further experiments were carried out with extracts from HeLa cells. In this case, only the mRNA of ARPC2, but not of MMP16, could be detected by RT-PCR (Figure 1D).

### Inhibition of translation by the MMP16 and ARPC2 RNA G-quadruplexes

**Figure 2.**
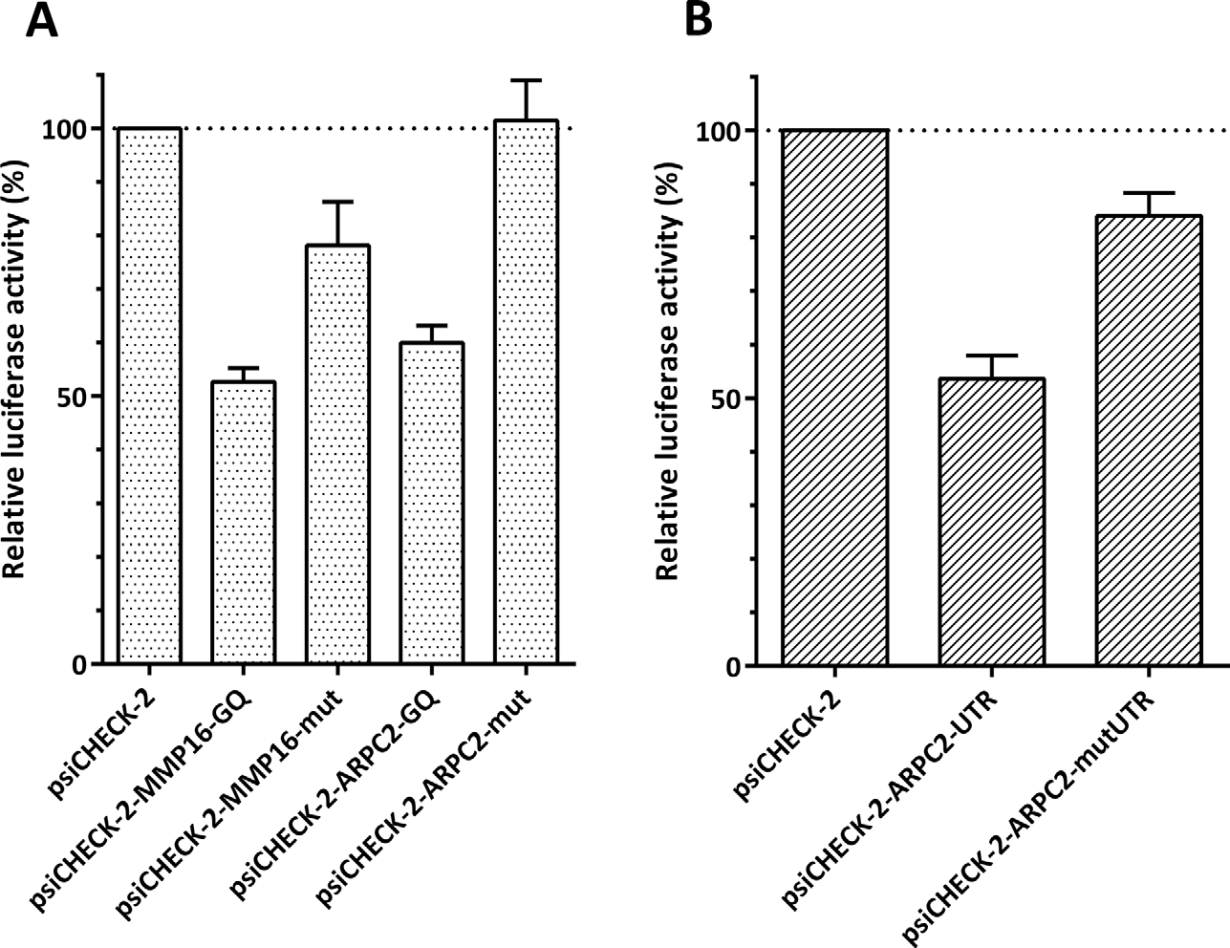
Inhibition of translation by the G-quadruplex motifs of MMP16 and ARPC2, as determined by dual luciferase assays. (**A**) Dual luciferase assays for the isolated G-quadruplex motifs and (**B**) for the full-length 5′-UTR of ARPC2. The ratio of *Renilla* and firefly luciferase activity is normalized to the value of the empty psiCHECK-2 vector. The G-quadruplex motif in the 5′-UTR of *Renilla* luciferase reduces enzyme synthesis. Values given are averages ± standard deviations of three experiments.

The next step was to analyze the inhibitory activity of the G-quadruplex motifs. These studies were carried out as described before for Zic-1 (11). Dual luciferase assays were performed with the psiCHECK-2 vector that allows simultaneous expression of *Renilla* and firefly luciferase from a single plasmid. DNA sequences encoding the RNA G-quadruplexes and their mutated controls, respectively, were cloned upstream of the *Renilla* luciferase. The vector constructs were transfected into HEK293 cells and 24 h after transfection cells were harvested. Dual luciferase measurements were carried out and the ratios of luciferase activity for the *Renilla* and firefly luciferase were normalized to the value obtained for the empty psiCHECK-2 vector. As can be seen in Figure 2A, both, the G-quadruplex motifs of MMP16 and ARPC2, repress translation by approximately 50% and 40%, respectively. The control for the MMP16 G-quadruplex also had a minor effect on the translation efficiency. The partial inhibition of translation by the G-quadruplexes is in the range of what was observed for other G-quadruplexes (2). Morris and Basu (14) found a slightly more pronounced repression of translation by the MMP16 G-quadruplex of 60%, which may be due to the fact that they used the full length 5′-UTR, while we only used the isolated G-quadruplex motif with a few flanking nucleotides.

For the less well characterized G-quadruplex of ARPC2, additional experiments were carried out with the full-length 5′-UTR to investigate whether the G-quadruplex also inhibits translation in its natural sequence environment. The results obtained with the full-length 5′-UTR (Figure 2B) were very similar to those described for the isolated G-quadruplex motif: the G-quadruplex sequence inhibited translation by approximately 50%, while a control with point mutations that prevent formation of the G-quadruplex only had a negligible effect. This finding supports the assumption that a G-quadruplex structure is formed in the 5′-UTR of the ARPC2 that inhibits translation.

### Identification of RNA G-quadruplex binding proteins by pull-down assays

The central aim of the present study was to isolate and identify proteins that bind to the RNA G-quadruplex motifs. Therefore, pull-down assays with whole cell extract and the G-quadruplex-forming RNA oligonucleotides or mutated control oligonucleotides were performed. Proteins were eluted with increasing KCl concentrations and further analyzed by SDS-PAGE. For MS, the following protein bands were chosen: (i) proteins that only bound to the G-quadruplex-forming RNA but not to the mutated sequences and (ii) proteins that bound to the mutated sequences of MMP16 and ARPC2.

**Figure 3.**
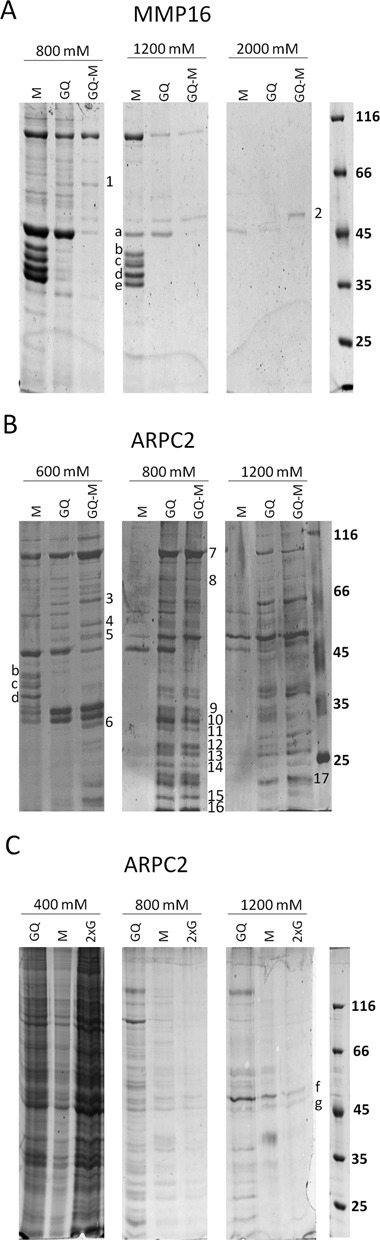
Pull-down assays. (**A**) and (**B**) G-quadruplex motifs of the MMP16 (A) and ARPC2 (B) mRNAs were coupled to streptavidin agarose beads. Whole-cell extract of HEK293 cells was added to the beads. After several washing steps, proteins were eluted with buffers containing the indicated K^+^-concentrations. Proteins were analyzed by SDS-PAGE. Proteins that bind exclusively to the G-quadruplex-sequence (Q) are indicated. GQ-M represents an independent experiment, in which the HEK293 extract was first incubated with the mutated oligonucleotide and then with the G-quadruplex-forming RNA. The indicated proteins were identified by MS-based peptide mass fingerprinting. (**C**) Pull-down assay with the additional ARPC2 control oligonucleotide with two G-stretches (ARPC2–2xG). 1. ME2 (66 kDa); 2. YB-1 (36 kDa); 3. U2AF65 (54 kDa); 4. hnRNPH (50 kDa); 5. YB-1 (36 kDa) hnRNPF (46 kDa); 6. RPS2 (32 kDa); 7. Nucl (76 kDa); 8. RBM14 (70 kDa); 9. SRSF1 (28 kDa); 10. SRSF1 (28 kDa) RPS6 (29 kDa); 11. RPL7 (29 kDa); 12. SRSF9 (26 kDa), RPS6 (29 kDa); 13. SRSF9 (26 kDa), RPL14 (23,5 kDa); 14. RPL10 (25 kDa) RPS9 (23 kDa); 15. RPL26 (17 kDa); 16. RPL27a (17 kDa); 17. RPS9 (23 kDa); a. Actin (42 kDa); b. hnRNPA3 (39 kDa); c. hnRNPA2B1 (37 kDa) and hnRNPA3 (39 kDa); d. hnRNPA2B1 (37 kDa); e. hnRNPA1 (39 kDa); f. YB-1 (36 kDA); g. YB-1/Actin (36 kDa, 42 kDa).

The experiments were conducted with extracts from HEK293 cells and were repeated three times. Figure 3 shows examples for pull-down assays with the MMP16 (A) and ARPC2 (B) G-quadruplex RNA oligonucleotides. Bands of proteins binding specifically to either of the G-quadruplex-forming RNAs were chosen for further analyses by MS. Bands appearing in both the G-quadruplex oligonucleotide (GQ) as well as the mutated control (M) lanes are most likely unspecific RNA binders.

To reduce proteins with a general capacity to bind to RNA, in an alternative approach, the cellular extracts were initially incubated with the control oligonucleotides. Subsequently, the depleted extracts were incubated with the G-quadruplex-forming oligonucleotides to pull down the specific binding partners. As can be seen in Figure 3 (lane GQ-M), these experiments mostly confirmed the proteins identified with the initial strategy.

Table 1 summarizes the proteins identified to specifically bind to G-quadruplex structures in the pull-down assays. The detailed MS data are given as supplementary (Supplementary Table S1). The number of times a protein was identified in repeated experiments is given. A much larger number of binding candidates were found for the ARPC2 G-quadruplex than for the MMP16 G-quadruplex. The higher number of identified proteins for the ARPC2 G-quadruplex could be a result of the variety of the G-quadruplex motifs putatively formed by ARPC2 compared to MMP16. As stated before, QGRS mapper analysis revealed that ARPC2 can form up to four variants of the G-quadruplex structure.

**Table 1.** Proteins identified to bind to the G-quadruplex motifs of the ARPC2 and MMP16 mRNA

	ARPC2 GQ		MMP16 GQ	
	Hek	HeLa	Hek	Hela
**Ribosomal Proteins**				
60S ribosomal protein L6 (RPL6^10^)	2^a^	1		
60S ribosomal protein L7 (RPL7^11^)	2	1		
60S ribosomal protein L10 (RPL10^14^)	2			
60S ribosomal protein L12 (RPL12)		1		
60S ribosomal protein L14 (RPL14^13^)	1			
60S ribosomal protein L19 (RPL19)	1	1		
60S ribosomal protein L26 (RPL26^15^)	1			
60S ribosomal protein L27a (RPL27a^16^)	1			
40S ribosomal protein S2 (RPS2^6^)	1			
40S ribosomal protein S5 (RPS5)				1
40S ribosomal protein S6 (RPS6^12^)	1			
40S ribosomal protein S9 (RPS9^14, 17^)	2	1		
**Splicing Factors**				
Heterogeneous nuclear ribonucleoprotein F (hnRNPF^5^)	1			
Heterogeneous nuclear ribonucleoprotein H (hnRNPH^4^)	2			
Heterogeneous nuclear ribonucleoprotein U (hnRNPU)			2	1
Serine/arginine-rich splicing factor 1 (SRSF1^9, 10^)	3	1		
Serine/arginine-rich splicing factor 9 (SRSF9^12, 13^)	2	1		
Splicing factor U2AF (U2AF65^3^)	1	1	2	
**Others**				
Y-box-binding protein 3	1			
EF-hand domain-containing protein D2 (EFHD2)	2	1		
NAD-dependent malic enzyme, mitochondrial (ME2^1^)			3	
Nucleolin (Nucl^7^)	3	2		
Ras GTPase-activating protein-binding protein 2 (G3BP2)		1		
RNA binding protein 14 (RBM14^8^)	2			
Tropomyosin alpha 4 (TPM4)	1			

^a^The number of times a protein was identified is given. Experiments with HEK293 extracts were carried out three times; confirmatory experiments with HeLa extracts were performed twice.

The superscripts refer to the bands marked in Figure 3.

Several ribosomal proteins (RPs) were found to interact with either of the two G-quadruplexes. Further interaction partners belong to the class of proteins involved in splicing, including the serine/arginine-rich splicing factors (SRSF) 1 and 9, the splicing factor U2AF65 and the nuclease-sensitive element-binding protein 1 (YB-1). A distinct binder of the MMP16 G-quadruplex was the mitochondrial nicotinamide adenine dinucleotide (NAD)-dependent malic enzyme (ME2). Most of the identified proteins were specific for one of the two G-quadruplex structures. Only the splicing factors U2AF65 and YB-1 bound to both motifs. Further control experiments revealed that the latter candidate (YB-1) has the general capacity of binding to G-rich sequences (see below).

The next aim was to test whether the RNA G-quadruplex-protein interaction is cell-type specific. To this end, the experiments were repeated with HeLa cells. The experiments with HeLa cells were repeated twice. As can be seen in Table 1, several binding proteins such as nucleolin and U2AF65 identified in the experiments with extracts from HEK293 cells were confirmed for the HeLa extracts and only one additional RP (RPS5) was newly identified to bind to the MMP16 G-quadruplex. Thus, similar interactions presumably occur in different cell types.

We also identified the proteins represented by some particularly prominent bands in the control oligonucleotides by MALDI-TOF. Interestingly, all of the identified proteins belong to the class of hnRNPs, and they were similar for both controls: hnRNP A3, A2/B1, A1. None of these proteins bound to the G-quadruplex-forming oligonucleotides. Only actin was identified as a protein that binds to the G-quadruplex motifs as well as to the controls. This finding is likely to reflect the high abundance of this cytoskeleton protein, rather than specific binding.

The experiments described so far discriminate between proteins binding to sequences that are capable of forming G-quadruplex structures and those with a general tendency to bind to RNA. To exclude proteins that bind to G-rich sequences, additional experiments were performed with a second control sequence with a higher guanine content and two G-stretches (ARPC2–2xG). As can be seen in Figure 3C, only very few proteins bound to this additional control at higher salt concentrations. The proteins of the most prominent bands marked as f and g, respectively, were identified as YB-1 and YB-1 in complex with actin. YB-1 thus does not seem to bind specifically to G-quadruplex structures, but rather recognizes G-rich sequences.

### Characterization of interactions between RNA G-quadruplexes and proteins

The binding parameters for U2AF65, nucleolin, SRSF1 and EFHD2 were determined by SPR spectroscopy. The proteins were recombinantly expressed with a His-tag in *E. coli* and purified by Ni-NTA-agarose affinity chromatography. Subsequently, the proteins were immobilized on a CMD sensor chip by amine coupling. Binding of the RNA oligonucleotides to the proteins was measured.

**Figure 4.**
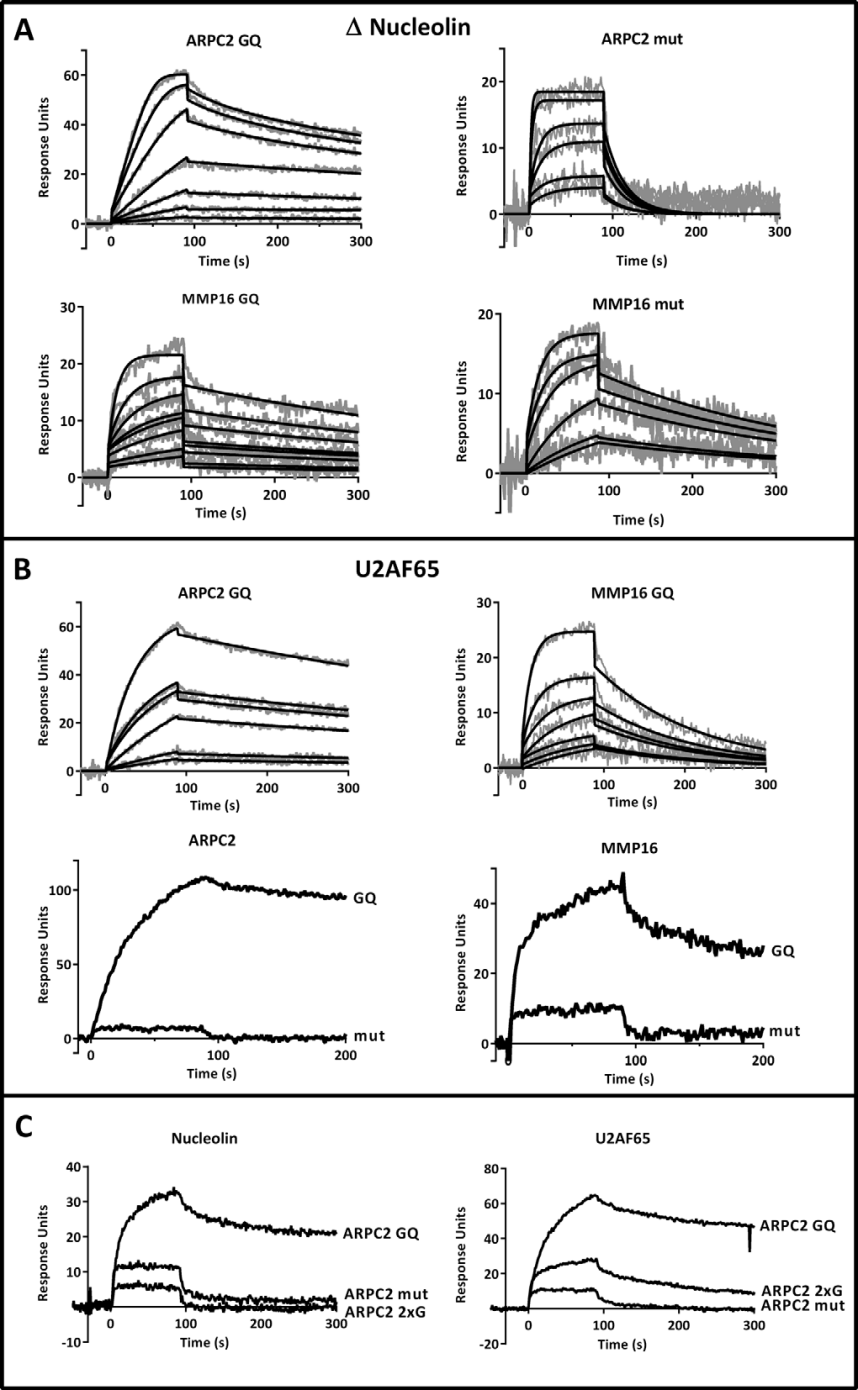
Analysis of protein-RNA interactions by SPR spectroscopy. (**A**) Binding parameters for Δnucleolin to the ARPC2 and MMP16 G-quadruplex and the mutated sequence were analyzed in the concentration range of 1–20 nM and 10–1000 nM, respectively. Data were fitted to 1:1 binding with mass transfer. Sensorgrams are shown in gray and the respective fits in black. (**B**) Binding of U2AF65 to 100 nM of the ARPC2 and MMP16 G-quadruplex oligonucleotides and experiments with the respective controls. (**C**) Additional experiments with the G-rich control oligonucleotides ARPC2 2xG in comparison to the G-quadruplex and the exhaustively mutated oligonucleotide.

Since full-length nucleolin is not soluble when heterologously expressed in *E. coli*, a shortened version was expressed lacking the N-terminal domain (residues 1–283). This protein, denoted as Δnucleolin, is composed of all four RBDs and the C-terminal domain. SPR measurements were carried out with Δnucleolin and a dissociation constant (*K*_D_) of 0.6 nM was determined for the ARPC2 G-quadruplex, while binding to the control oligonucleotide was more than 100-fold weaker, with a *K*_D_ of 83 nM (Table 2 and Figure 4A). Nucleolin was identified as a binding partner of the ARPC2 G-quadruplex, but to test if there is a general interaction with G-quadruplexes, we also examined its affinity for the MMP16 sequences. Surprisingly, here the protein bound tightly to the control as well as to the G-quadruplex oligonucleotide of MMP16 (Figure 4A), indicating that nucleolin is able to recognize diverse nucleic acid motifs.

**Table 2.** Association (*k*_a_) and dissociation (*k*_d_) rates and dissociation constants (K_D_) determined by SPR spectroscopy; n. d.: not determined due to too weak interactions

Ligand	Analyte	*k*_a_ (M^−1^s^−1^) x 10^6^	*k*_d_ (s^−1^) x 10^−3^	*K*_D_ (nM)	χ^2^
ΔNucleolin	ARPC2 GQ	8.7	5.1	0.6	0.96
	ARPC2 mut	0.5	41.4	83	1.61
	MMP16 GQ	0.92	1.9	2	0.79
	MMP16 mut	6	3.6	0.6	0.94
U2AF65	ARPC2 GQ	1.1	1.2	1	0.9
	ARPC2 mut			n. d.	
	MMP16 GQ	1.6	8.1	5	0.73
	MMP16 mut			n. d.	

The splicing factor U2AF65 was next investigated as a candidate for specific binding to G-quadruplexes, as it was found to bind to both G-quadruplexes in the pull-down assays. SPR measurements confirmed that it binds to the ARPC2 and MMP16 G-quadruplexes, while it does not interact with the mutated controls (Figure 4B). Dissociation constants of 1 nM for the ARPC2 G-quadruplex and 5 nM for the MMP16 G-quadruplex were determined (Table 2). Binding to both mutated control sequences was so weak that it was impossible to determine a *K*_D_. U2AF65 can thus be considered to bind specifically to RNA G-quadruplex structures, while it does not seem to be specific for a certain G-quadruplex motif.

Additional experiments were carried out with the above-described, guanine-rich control oligonucleotide ARP2–2xG to confirm that the proteins interact specifically with the G-quadruplex structures. As can be seen in Figure 4C, ARPC2 2xG behaves similar to the fully mutated sequence, i.e. nucleolin and U2AF65 only bind to a sequence that is capable of forming a G-quadruplex structure, but not to a G-rich sequence. This finding further supports the hypothesis that the identified proteins bind to the G-quadruplex structure.

**Figure 5.**
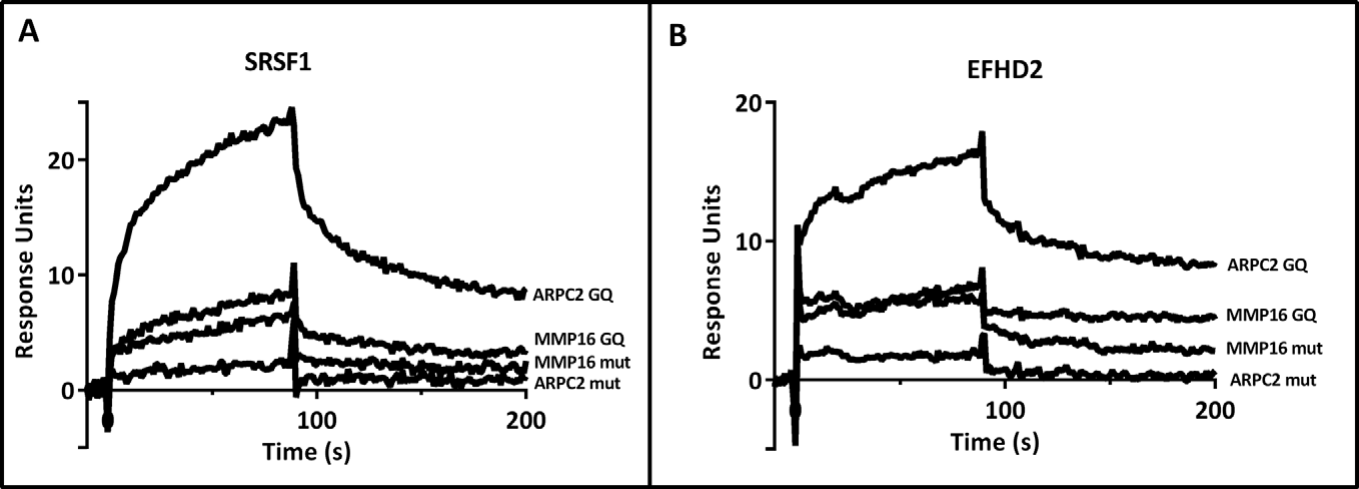
Analysis of protein-RNA interactions by SPR spectroscopy. Binding of SRSF1 (**A**) and EFHD2 (**B**) to 100 nM of the G-quadruplex and mutated control sequences of ARPC2 and MMP16.

According to the pull-down assays, SRSF1 and EFHD2 were two prominent and specific interaction partners of the ARPC2 G-quadruplex. Since both proteins rapidly lost activity when coupled to the sensor chip, only a qualitative comparison of the binding to either of the two G-quadruplexes and the respective control sequences was conducted. In the assay, the mutated oligonucleotides were injected first to counteract the effect of the activity loss. Both proteins bound much stronger to the ARPC2 G-quadruplex than to the MMP16 G-quadruplex or to any of the control sequences (Figure 5). These proteins can therefore be regarded to be highly specific for a certain G-quadruplex motif.

## DISCUSSION

G-quadruplex motifs in RNA have only come into the focus of research in recent years (2,6,7,9). They have been reported to be involved in regulation of translation and to have a function in splicing (9). It is tempting to speculate that the stability and activity of G-quadruplexes might be modulated by cellular factors such as proteins. Indeed, G-quadruplexes have a great potential to bind proteins with high affinity and specificity as shown for aptamers, which often form a G-quadruplex structure. The identification and characterization of interactions between RNA G-quadruplexes and cellular proteins will help increasing our understanding of the physiological functions of these structural elements (6,8). We present the first systematic approach to identify proteins that bind to G-quadruplex elements in 5′-UTRs of mRNAs using pull-down assays and MALDI-TOF MS. Extensive controls were carried out to distinguish between proteins that bind specifically to G-quadruplex structures and those that have a general capacity to interact with RNA or with guanine-rich sequences.

In the present study, we focused on G-quadruplexes located in the 5'-UTR, which have been reported to modulate translation without affecting the mRNA level (8,22). Indeed, several of the identified proteins are involved in translation. A number of RPs like RPS5, RPS6 and RPS9 were found to bind to the ARPC2 or MMP16 G-quadruplex. All three are part of the 43S pre-initiation complex that moves along the mRNA when scanning for the start codon. For G-quadruplexes located in the 5'-UTR it can be assumed that, depending on the position, the quadruplex may inhibit recognition of the cap or start codon or the scanning of the 40S ribosomal subunit for the translational start (42). This could be due to steric blockage of the G-quadruplex structure itself or because of proteins binding to the quadruplex.

Some of the identified proteins are known to be involved in the formation of ribonucleoprotein complexes like the nucleolin-binding ribonucleoprotein complex (43) consisting, among others, of nucleolin, hnRNPs, RPL6, RPL7, RPL10, RPL27a, RPS6 and RPS9, all of them found as binders of the ARPC2 G-quadruplex. Without further analyses, it is difficult to differentiate between proteins interacting directly with the RNA G-quadruplex and proteins which are part of multimeric ribonucleoprotein complexes. In addition, YB-1 also bound to a G-rich sequence that cannot fold into a G-quadruplex structure. It is therefore likely that it recognizes stretches of guanines rather than G-quadruplexes.

To gain a deeper insight, Δnucleolin was heterologously expressed in *E. coli* and purified via affinity chromatography. Binding constants were determined by SPR to be in a low nanomolar range for the ARPC2 G-quadruplex (Table 2). The strength of binding is thus in the typical order of magnitude of strong biological interactions, indicating that at least nucleolin binds directly to the ARPC2 G-quadruplex.

A comparison of the binding affinities of Δnucleolin to the ARPC2 G-quadruplex and the mutated sequence revealed binding constants of 0.6 nM and 83 nM, respectively (Table 2). Thus, binding of nucleolin to the ARPC2 G-quadruplex is more than 100-fold stronger and seems to be dependent on the formation of the quadruplex structure. The binding constant is in good accordance with nanomolar values described for binding of nucleolin to a G-quadruplex structure formed by ribosomal DNA (44). However, a comparison of the binding constants of Δnucleolin to the MMP16 G-quadruplex and its mutated sequence revealed values of 2 and 0.6 nM, respectively (Table 2). An even stronger binding of Δnucleolin to the mutated sequence was determined, although it is not able to form the 3D structure of G-quadruplexes. There are various nucleolin binding motifs described in the literature, such as G-rich consensus motifs, a loop consisting of the nucleotides UCCCGA, AU-rich elements and G-quadruplexes (45–48). This diversity may result from the different RBDs of nucleolin. It is composed of an acidic N-terminus, four central RBDs and a C-terminal RNA-binding motif, the RGG domain (49). The RBDs 3 and 4, as well as the RGG domain were found to be required for interaction between nucleolin and G-quadruplexes (50).

Members of the family of hnRNPs (hnRNP F, H and U) were also identified as potential RNA G-quadruplex-binding proteins. HnRNPs belong to a group of multifunctional proteins involved in all important steps of RNA processing, including maturation and transport of transcripts or translational regulation (51). The hnRNPs F and H were shown to specifically bind to poly(G) tracts (52). In a mechanistic study, Samatanga et al. (53) described that the third RBD of hnRNP F bound exclusively to single-stranded G-tracts and does not recognize the G-quadruplex. However, in our control experiments with the single-stranded G-rich stretch that cannot form a G-quadruplex, we did not observe interactions with hnRNPs. Samatanga et al. propose a model according to which hnRNP F binds co-transcriptionally to mRNA, thereby preventing G-quadruplex formation. A mechanism assuming that hnRNP F and H bind to G-quadruplexes and dissolves the 3D structure, however, is also imaginable. In both cases, the interaction between the proteins and the G-quadruplexes results in enhanced translation. Less is known about the function and properties of hnRNP U, but the protein was reported to bind AT-rich DNA elements (54), while we found an interaction with a G-quadruplex.

As outlined before, G-quadruplexes located in the 5'-UTR are thought to play a role in the regulation of translation. However, for ARPC2 and MMP16 various additional proteins were identified by MS which are linked to splicing processes (Table 1). While an involvement in the splicing process of G-quadruplexes located in the coding region was described in previous publications (55–57), a G-quadruplex-dependent splicing in the 5'-UTR has not been shown so far. The identification of proteins involved in splicing or alternative splicing like U2AF65, serine/arginine-rich (SR) proteins or hnRNPs also suggests a function of G-quadruplexes in 5'-UTR splicing.

A role of G-quadruplexes in alternative splicing was shown for the tumor suppressor gene *TP53* mRNA, the BACE1 (β-site amyloid precursor protein cleaving enzyme 1) mRNA and the *FMR1* (fragile X mental retardation) mRNA (55–58). In the mRNA of *TP53* the G-quadruplex structure in intron 3 modulated the splicing of intron 2 (55). Treatment of a lymphoblastoid cell line with a synthetic ligand that binds to single-stranded G-quadruplex structures altered the ratio of two different splice forms. Moreover, a G-quadruplex in an exon of the mRNA of BACE1 was found to be involved in controlling splice site selection (56). In the *FMR1* mRNA, the G-quadruplex motif functioned as an exonic splicing enhancer that increased the inclusion of an alternatively spliced exon (57). Exonic splicing enhancers are sequences within exons that promote constitutive as well as regulated splicing. It is thought that splicing factors like U2AF65 or SRSF1 are involved in the recognition of these sequences (58).

An interaction between SRSF1 or U2AF65 and G-quadruplexes as demonstrated in the present study has not been shown before. SR proteins are a conserved family of proteins involved in constitutive, as well as in alternative, splicing by binding to exonic and intronic sites to enhance exon recognition (59). SR proteins bind to exonic splicing enhancers with their N-terminal RNA-recognition motifs. Two SR proteins were found to interact with the G-quadruplex in the 5′-UTR of ARPC2 (Table 1). Relatively little is known about SRSF9, but the protein was described to function as a repressor of 3′ splice site-utilization (60).

The *U2AF65* gene encodes the large, 65 kDa subunit of the U2 auxiliary factor (U2AF). Originally, U2AF65 was shown to be involved in recognition of 3′ splice sites by binding to the polypyrimidine tract located between the branch point sequence and the AG dinucleotide. It was also identified as a major triplex DNA-binding protein by Nelson and colleagues (61), who speculated that U2AF65 could be able to bind multi-stranded nucleic acid structures like G-quadruplexes, R-loops or D-loops. Our SPR analysis confirmed the strong interactions between U2AF65 and the RNA G-quadruplexes of both ARPC2 and MMP16. We determined dissociation constants of 1 and 5 nM, respectively, while binding to the control sequences was very weak, suggesting a biologically relevant binding to both G-quadruplex structures.

While the capacity to interact with nucleic acids or G-quadruplexes has previously been described for a number of the identified proteins, no such properties have been ascribed to EFHD2 and ME2. EFHD2 is a calcium-binding protein containing three binding sites for SH3 domain-containing proteins, two EF-hand domains, and a coiled-coil domain at the C-terminus. The protein has been shown to be involved in signaling in immature B cells (62,63) and was now found as binding partner of the ARPC2 G-quadruplex (Table 1). ME2 was identified as a pronounced interaction partner of the G-quadruplex of MMP16. It catalyzes the oxidative decarboxylation of malate to pyruvate in the mitochondrion (64). RNA-binding activity was shown neither for EFHD2 nor for ME2 before and might therefore indicate a previously unknown function for both proteins.

For ME2 it may be speculated that interactions with G-quadruplexes may have an impact on the regulation of metabolic pathways. A search for potential G-quadruplex-forming sequences in the 5'-UTR of ME2 revealed four hits. Thus it is conceivable that ME2 regulates its own translation by binding to these G-quadruplex structures. Such a negative feedback loop was shown before for FMRP and the G-quadruplex located in its own mRNA, where it can regulate its splicing (65).

While U2AF65 was binding to both G-quadruplex motifs, all other candidates were specific for either of the two structures, suggesting two different binding mechanisms. The universal binders can be assumed to recognize the G-quadruplex structures, while the specific binders may interact with the loop sequence, which is variable in different G-quadruplexes. Pull-down experiments were carried out with extracts from two cell lines, HEK293 and HeLa cells, and similar results were obtained in both cases. The interactions thus do not seem to be specific for a certain cell type.

In summary, we have isolated proteins that bind to G-quadruplex motifs in the 5′-UTR of mRNAs for the first time using a systematic screening approach. A number of proteins known to regulate translation such as RPs or nucleolin were identified verifying the role of G-quadruplexes in translation. Additionally, proteins involved in splicing or alternative splicing processes have been detected by MS, indicating a role of G-quadruplexes in 5'-UTR splicing. Moreover, we identified proteins that have previously not been associated with interactions to nucleic acids. Most of the identified proteins are specific for either of the two investigated G-quadruplex motifs, while some exceptions seem to have a more general capacity to bind to these structural elements. The determined dissociation constants in the low nanomolar range indicate strong interactions with biological relevance. As an important control, we demonstrate that the identified proteins do not bind to G-rich sequences in general, but only to sequences that are capable of forming a G-quadruplex structure. It will now be necessary to elucidate how binding of the proteins influences stability of the G-quadruplexes and finally modulates translational efficiency or alternative splicing processes to gain deeper insight into the biological relevance of protein-RNA G-quadruplex interactions.

## SUPPLEMENTARY DATA

Supplementary Data are available at NAR Online.

SUPPLEMENTARY DATA
