# Supplementary material for: Identification and characterization of RNA guanine-quadruplex binding proteins
Source: Nucleic Acids Res. 2014 Apr 25;42(10):6630–44. doi: 10.1093/nar/gku290 (PMC4041461; doi:10.1093/nar/gku290)
Supplement: SUPPLEMENTARY DATA [file supp_42_10_6630__index.html]

Identification and characterization of RNA guanine-quadruplex binding proteins — Identification and characterization of RNA guanine-quadruplex binding proteins — SUPPLEMENTARY DATA 

# Identification and characterization of RNA guanine-quadruplex binding proteins

## SUPPLEMENTARY DATA

**Files in this Data Supplement:**

- SUPPLEMENTARY DATA
