# Supplementary material for: Identification and characterization of RNA guanine-quadruplex binding proteins
Source: Nucleic Acids Res. 2014 Apr 25;42(10):6630–44. doi: 10.1093/nar/gku290 (PMC4041461; doi:10.1093/nar/gku290)
Supplement: SUPPLEMENTARY DATA [file supp_gku290_nar-02316-f-2013-File007.pdf]

Table S1. Mass spectrometry data of proteins identified to bind to G-quadruplex sequences or to control sequences in this work. Database searches were performed using Mascot against the SwissProt database. The search was restricted to human sequences.

Identified Protein	SwissProt Accession code	Spot #	Apparent MW (kDa)	Theoretical MW (kDa)	Mass finger-print data Expect value	Peptide tolerance (PMF, +/- ppm)	Assigned Peptides	Sequence Coverage (%)	MS+MS/MS Combined (Protein summary deprecated) Expect value	Number of MS/MS	Selected masses for MS/MS
**Ribosomal Proteins**											
60S ribosomal protein L6	RL6_HUMAN	10	35	33	0.002	75	9	23	6.60E-07	1	1158.68
					0.001	150	13	29			
							1*		2.00E-10	1	2509.20
60S ribosomal protein L7	RL7_HUMAN		30	29	2.0E-06	75	11	35			
		11			8.3E-06	100	12	39			
					0.00011	75	9	32			
60S ribosomal protein L10	RL10_HUMAN		25	25	0.0036	75	8	27			
		14			0.003	60	10	30			
60S ribosomal protein L12	RL12_HUMAN		20	18			1*		0.1	2	714.31, 1685.89
60S ribosomal protein L14	RL14_HUMAN		25	24	0.032	75	9	32			
60S ribosomal protein L19	RL19_HUMAN		25	24	0.00019	125	12	40	0.00019	3	1065.41, 1454.54, 1942.75
					5.6E-06	75	15	32			
60S ribosomal protein L26	RL26_HUMAN	15	17	17		75			4.0E-10		955.44, 1078.62
60S ribosomal protein L27a	RL27A_HUMAN	16	16	17	0.0042	75	8	41			
40S ribosomal protein S2	RS2_HUMAN	6	30	32	2.4E-06	50	11	28			
40S ribosomal protein S5	RS5_HUMAN		23	23	0.00011	75	11	31	2.6E-07	1	1100.60
40S ribosomal protein S6	RS6_HUMAN		28	29	0.023	100	9	30			
40S ribosomal protein S9	RS9_HUMAN		23	23	5.1E-07	75	14	45			
					0.003	60	10	30			
					1.0E-12	75	17	48			
		17			4.0E-08	50	12	42			

**Splicing Factors**											
Heterogeneous nuclear ribonucleoprotein F	HNRPF_HUMAN	5	48	46	2.0E-08	75	15	39			
Heterogeneous nuclear ribonucleoprotein H	HNRH1_HUMAN	4	50	50	1.0E-08	125	17	42			
					5.1E-07	70	18	42			
Heterogeneous nuclear ribonucleoprotein U	HNRPU_HUMAN		116	91	1.6E-07	75	27	27			
					0.00072	75	17	20	n.s.	2	976.44, 1198.69
					0.00042	70	12	14			
Serine/arginine-rich splicing factor 1	SRSF1_HUMAN		30	28	0.11	50	10	46			
		9			0.017	100	10	39			
		10			4.2E-06	100	11	40			
					6.9E-06	75	12	50			
Serine/arginine-rich splicing factor 9	SRSF9_HUMAN		25	26	0.066	125	15	55	8.1E-12	3	1065.41, 1454.54, 1942.75
					6.7E-05	75	13	43			
		12			0.0002	75	11	36			
		13			0.0099	75	8	28			
Splicing factor U2AF	U2AF2_HUMAN		66	54	6.4E-13	75	18	41	4.1E-17	4	960.50, 1285.72, 1909.06, 1923.00
					3.2E-08	75	12	39			
					2.2E-05	70	12	31	2.0E-11	3	955.49, 1368.60, 1909.02
		3			0.0012	75	10	21			
**Others**											
EF-hand domain-containing protein D2	EFHD2_HUMAN		32	27	6.4E-10	75	15	52			
					0.0002	50	12	42			
					1.3E-05	100	12	46			
Ras GTPase-activating protein-binding protein 2	G3BP2_HUMAN				2.0E-07	54	16	32			

NAD-dependent malic enzyme, mitochondrial	MAOM_HUMAN			66	0.0022	75	11	23	1.0E-12	4	960.50, 1285.72, 1909.06, 1923.00
					0.00032	75	11	24			
		1			4.0E-08	75	16	27			
Nucleolin	NUCL_HUMAN		100	77	4.7E-06	75	21	27			
					1.3E-10	100	23	28			
		7			8.1E-11	75	20	25			
					2.6E-12	75	24	35			
					2.5E-09	70	20	27			
RNA binding protein 14	RBM14_HUMAN		70	70	1.0E-12	75	23	34			
		8			2.0E-07	75	12	22			
Tropomyosin alpha 4	TPM4_HUMAN		35	29	1.3E-06	75	16	45			
Y-box-binding protein 3	YBOX3_HUMAN		60	40	3.2E-07	75	13	31	8.3E-12	1	1559.80
**Proteins binding to controls**											
Actin, aortic smooth muscle	ACTA_HUMAN	g	45	42	0.067	120	6	18	0.00038	1	1198.70
Actin, cytoplasmic 1	ACTB_HUMAN	a	45	42	6.4E-11	75	20	60			
Actin, cytoplasmic 2	ACTG_HUMAN	a	45	42	6.4E-11	75	20	60			
Heterogeneous nuclear ribonucleoprotein A1	ROA1_HUMAN	e	34	39	5.1E-16	75	23	50			
Heterogeneous nuclear ribonucleoproteins A2/B1	ROA2_HUMAN	c	38	38	1.6E-12		22	63			
Heterogeneous nuclear ribonucleoproteins A2/B1	ROA2_HUMAN	d	36	38	1.0E-10	50	17	43			
Heterogeneous nuclear ribonucleoprotein A3	ROA3_HUMAN	b	40	40	1.0E-07	75	17	46			
		c	38	40	0.00066	75	12	36			
Nuclease-sensitive element-binding protein 1	YBOX1_HUMAN	2	48	36	2.5E-11	75	17	37			
		f			2.6E-08	75	12	23			
		g			4.0E-07	120	11	25	2.00E-07	1	1198.70

PMF, peptide mass fingerprint; * Proteins were identified by unique peptide sequences determined by MALDI-TOF-MS/MS experiments
